# Spontaneous Bladder Wall Rupture Due to Emphysematous Cystitis

**DOI:** 10.5334/jbr-btr.1151

**Published:** 2016-10-18

**Authors:** Pieter Roels, Karel Decaestecker, Pieter De Visschere

**Affiliations:** 1 Ghent University Hospital, BE

**Keywords:** Bladder rupture, Bladder perforation, Emphysematous cystitis

## Case History

**Figure 1:**
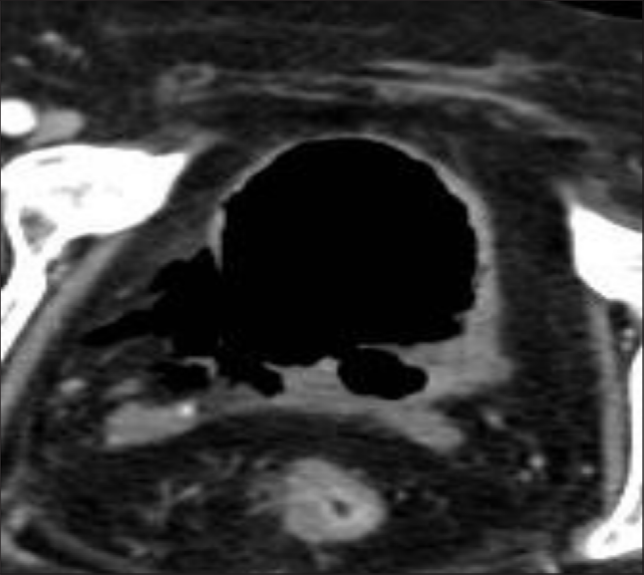
Axial unenhanced CT scan of the abdomen shows a disruption of the bladder wall at the right ­posterolateral side.

**Figure 2:**
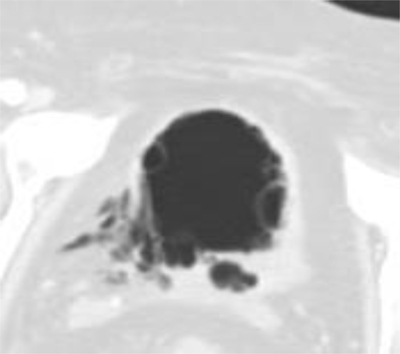
Same Axial unenhanced CT-scan image as above in Lung Window shows fibrotic tissue at the disrupton site. There are multiple air-filled submucosal blebs of the bladderwall suggesting an emphysematous cystitis.

**Figure 3:**
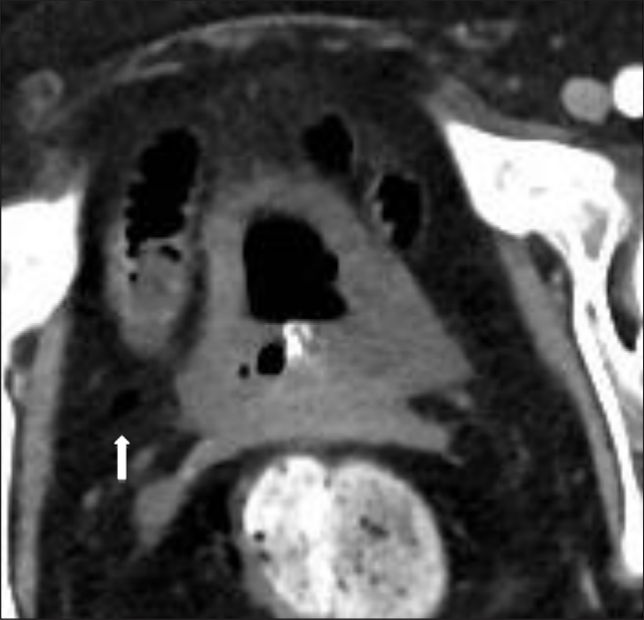
One-week later follow-up CT was performed, this showed a spontaneous healing of the bladder wall with only a small residual focus of free air in the Retzius space on the right (white arrow).

A 74-year-old woman presented with a vague suprapubic pain and progressive deterioration of her general condition. CT examination showed a partially air filled bladder with disruption of the bladder wall on the right posterolateral side. There was no intra-abdominal free air or free fluid. The bladder was slightly thickened (Figure 1). In Lung window, multiple air-filled mucosal bullae of the bladder were demonstrated (Figure 2). The latter is suggestive of an emphysematous cystitis (EC). Patient was treated conservatively with placement of a Foley catheter and with IV antibiotics. One week later, follow-up CT was performed; this showed a spontaneous healing of the bladder wall with only a small residual focus of free air in the Retzius space (Figure 3, arrow).

## Comment

Most cases of bladder rupture are traumatic. Spontaneous rupture is much more rare. Risk factors of spontaneous bladder wall rupture are urine retention due to diabetes, neurologic bladder, prostatic hyperplasia, history of pelvic radiotherapy or recurrent infections. There are also cases reported of bladder rupture due to binge drinking and due to obstructed labour. Bladder diverticula are an extra risk factor because they are responsible for an additional weakness of the bladder wall. In our case, an emphysematous cystitis caused bladder wall weakening and secondary rupture.

Patients with bladder rupture can present with haematuria, anuria, suprapubic pain and fever. Rupture can be divided into intraperitoneal (10–20% of cases) or extraperitoneal (80–90% of cases) depending on the site of disruption. Intraperitoneal rupture is associated with an acute abdomen. Our patient had an extraperitoneal rupture that was limited to perivesical space.

Intraperitoneal rupture needs open surgery while most extraperitoneal rupture can be treated conservatively with placement of Foley catheter. Cystography can identify the type and extend of rupture. Nowadays, CT cystography is the procedure of choice, especially in an emergency setting.

Emphysematous cystitis (EC) is a relatively rare variant of a urinary tract infection caused by gas-forming microbes. E. Coli is by far the most common isolated urinary pathogen, as in our case. It is typically seen in elderly women (60–70 years of age). Risk factors are the same as in uncomplicated cystitis like diabetes mellitus, neurogenic bladder and urinary stasis due to bladder outlet obstruction. The clinical course can range from asymptomatic to severe sepsis. Abdominal pain, dysuria and haematuria are most common. EC can be diagnosed radiologically by conventional radiography as a curvilinear increased radiolucency in the bladder wall. Intraluminal gas in the bladder will be seen as a dynamic air-fluid level. The mucosal surface may have a cobblestone or beaded ‘necklace’ appearance reflecting the irregular thickening produced by submucosal blebs as seen at direct cystoscopy. In our case we could also see these blebs on CT using a lung window. Furthermore, CT is also useful to rule out associated findings such as pyelonephritis, intra-abdominal abscess, malignancy or other causes of intraluminal gas such as an enterovesical fistula. EC may be suggested on ultrasound when dirty shadowing of the bladder wall is seen. Therapy of EC is similar as treatment of extraperitoneal bladder rupture: hospitalization with intravenous antibiotics, bladder drainage with Foley catheter and handling of predisposing conditions.

## Conclusion

Spontaneous rupture of the bladder wall is rare, especially due to emphysematous cystitis. Clinical presentation can range from asymptomatic to severe sepsis, so a high degree of suspicion during diagnosis is essential, especially in patients with some cause of urine retention. Findings at initial CT can be negative, so CT cystography is the modality of choice to rule out a tear and allow accurate diagnosis of the type of bladder wall rupture.
